# Dysbiotic Gut Microbiota-Derived Metabolites and Their Role in Non-Communicable Diseases

**DOI:** 10.3390/ijms242015256

**Published:** 2023-10-17

**Authors:** Jian Tan, Jemma Taitz, Ralph Nanan, Georges Grau, Laurence Macia

**Affiliations:** 1 Charles Perkins Centre, The University of Sydney, Sydney, NSW 2006, Australia; jian.tan@sydney.edu.au (J.T.); jemma.taitz@sydney.edu.au (J.T.); ralph.nanan@sydney.edu.au (R.N.); 2 School of Medical Sciences, Faculty of Medicine and Health, The University of Sydney, Sydney, NSW 2006, Australia; georges.grau@sydney.edu.au; 3 Sydney Medical School and Charles Perkins Centre Nepean, The University of Sydney, Sydney, NSW 2006, Australia; 4 Sydney Cytometry, The Centenary Institute and The University of Sydney, Sydney, NSW 2006, Australia

**Keywords:** gut microbiota, dysbiosis, metabolites, bacteria product, extracellular vesicles

## Abstract

Dysbiosis, generally defined as the disruption to gut microbiota composition or function, is observed in most diseases, including allergies, cancer, metabolic diseases, neurological disorders and diseases associated with autoimmunity. Dysbiosis is commonly associated with reduced levels of beneficial gut microbiota-derived metabolites such as short-chain fatty acids (SCFA) and indoles. Supplementation with these beneficial metabolites, or interventions to increase their microbial production, has been shown to ameliorate a variety of inflammatory diseases. Conversely, the production of gut ‘dysbiotic’ metabolites or by-products by the gut microbiota may contribute to disease development. This review summarizes the various ‘dysbiotic’ gut-derived products observed in cardiovascular diseases, cancer, inflammatory bowel disease, metabolic diseases including non-alcoholic steatohepatitis and autoimmune disorders such as multiple sclerosis. The increased production of dysbiotic gut microbial products, including trimethylamine, hydrogen sulphide, products of amino acid metabolism such as p-Cresyl sulphate and phenylacetic acid, and secondary bile acids such as deoxycholic acid, is commonly observed across multiple diseases. The simultaneous increased production of dysbiotic metabolites with the impaired production of beneficial metabolites, commonly associated with a modern lifestyle, may partially explain the high prevalence of inflammatory diseases in western countries.

## 1. Introduction

A finely-tuned interaction between the gut microbiota and the host is important for supporting the physiology and immunity of the host across its entire lifespan [1]. Disruption to this interaction, resulting from antibiotics usage or poor diet consumption, is linked to disturbances in both the composition and function of the gut microbiota. This disturbance, known as dysbiosis, is believed to set the scene for the development of chronic diseases [2,3].

The gut microbiota communicates with and exerts its influence on the host through multiple pathways. It does so via the binding of conserved pathogen-associated molecular patterns (PAMPs) to pattern recognition receptors expressed mostly on innate immune cells, such as epithelial cells and macrophages [4]. These PAMPs are typically found on the surface of bacteria but also on the surface of extracellular vesicles produced by bacteria [5]. The other major pathway of communication is the production of metabolites such as short-chain fatty acids (SCFA). SCFA can activate G protein-coupled receptors expressed by cells in the gut or in other organs, explaining the local [6,7] and systemic effects of the gut microbiota on the host [8,9]. The effects of SCFA include inducing tolerogenic dendritic cells and regulatory T cells (Treg) [10], which offer protection from the development of allergies [6], or activating the NLRP3 inflammasome in colonic epithelial cells, offering protection from colitis and colorectal cancer [7]. Other metabolites, such as secondary bile acids, have also been shown to support Treg development by activating nuclear receptors such as the vitamin D receptor [11]. Finally, gut microbiota-derived SCFA can also directly enter cells and hijack their metabolic pathways, supporting the development of anti-inflammatory IL-10-producing B cells both in mice and humans, with benefits to those who suffer from arthritis [12].

Currently, the focus of most research has been on understanding and harnessing the health benefits of the gut microbiota. This includes identifying and characterizing prebiotics (foods that beneficially reshape the gut microbiota), probiotics (beneficial gut bacteria) or postbiotics (beneficial bacterial by-products) and their mechanisms of action. Recent work has, however, highlighted the need to better understand the potential detrimental effects of gut bacterial by-products on disease development and/or in response to therapies [13]. The gut microbiota has the potential to not only contribute to or perpetuate diseases such as periodontitis, colitis, endometriosis, tumorigenesis, but can also impair the efficacy of treatments. Gut bacterial by-products could thus be used as biomarkers for better diagnostics [14] or to predict treatment efficacy. The aim of this review is to provide an overview of the current knowledge on the link between bacterial metabolites associated with dysbiosis, non-communicable disease development and response to therapies.

## 2. Cardiovascular Diseases and Periodontitis

Cardiovascular diseases (CVD) are the leading cause of death worldwide and are commonly associated with obesity or metabolic disorders. However, increasing numbers of patients with CVD have no such associated disorders, suggesting that other lifestyle factors are involved. Recent evidence suggests that gut dysbiosis and gut bacterial metabolites may contribute to CVD [15]. These effects are not only attributed to bacteria in the colon but also to bacteria located in the oral cavity, with periodontitis being a major risk factor for CVD.

Among the detrimental metabolites, trimethylamine N-oxide (TMAO) has been well-studied in metabolic diseases and CVD. Trimethylamine (TMA) is produced by certain gut bacteria from dietary choline, L-carnitine, glycine and betaine. The majority of TMA is then oxidised by hepatocytes via flavin monooxygenases in the liver, producing TMAO while a small amount may be utilized by methanobacteria to generate methane and ammonia. The intake of TMAO precursors such as L-carnitine, present in red meat, is directly associated with TMAO level, which in turn has been identified as one of the strongest predictive risk factors for CVD [16]. GWAS studies have also demonstrated that gut bacteria-derived betaine is causally associated with increased risk of CVD [17]. This increased risk likely relates to the pro-inflammatory functions of TMAO. TMAO activates p38 MAPK signalling in vivo as well as in vitro in primary human endothelial and smooth muscle cells, which upregulates pro-inflammatory factors such as COX-2, IL-6, E-selectin and ICAM-1 [18]. TMAO also enhances trained immunity in endothelial cells by increasing endoplasmic reticulum stress and metabolic reprogramming, which has been shown to induce a pro-inflammatory phenotype [19]. TMAO can also activate the NLRP3 inflammasome, leading to IL-1β secretion and endothelial cell dysfunction [20]. Consumption of a diet rich in TMAO precursors has also been shown to increase pro-inflammatory markers related to macrophage recruitment in aortic tissues [18]. On the other hand, this proinflammatory effect and increased activation of immune cells by TMAO may be beneficial in some contexts, such as in cancer. TMAO has been shown to improve anti-PD1 and/or anti-Tim3 immune checkpoint blockade in pancreatic ductal adenocarcinoma by potentiating IFN-g response [21] and activating CD8 T cell antitumor response, as well as by directly inducing the pyroptosis of tumour cells by increasing endoplasmic reticulum stress in triple-negative breast cancer [22]. TMAO thus perpetuates inflammation via various pathways, which may be beneficial or detrimental depending on the disease context.

Products of microbial amino acid metabolism can also impact the host. Microbial metabolism of L-tryptophan and L-tyrosine produces indole and p-Cresyl respectively, which can be further metabolized in the liver into indoxyl sulphate and p-Cresyl sulphate. Indoxyl sulphate is a cardiotoxin and 7-week exposure to p-Cresyl sulphate or indoxyl sulphate has been shown to induce arterial calcification in mice [23]. Exposure has also triggered a prodiabetic state characterized by impaired glucose homeostasis and a decrease in GLUT1 expression [23]. Another gut bacteria-derived tryptophan metabolite, indole-3-propionic acid (IPA), has been shown to increase blood pressure in rats. The echocardiographic analysis revealed higher heart contractility in IPA treated rats and IPA modified cardiomyocytes metabolism in vitro [24]. However, IPA levels have been shown to be inversely correlated with atherosclerosis [25]. Microbial metabolism of another amino acid, phenylalanine, produces phenylacetic acid. Phenylacetic acid can be transformed into phenylacetylglutamine in the liver, a metabolite associated with CVD and its incidental, major and adverse cardiovascular events [26]. Phenylacetylglutamine contributes to CVD by acting on α2A, α2B, and β2-adrenergic receptors on platelet cells, leading to an activated phenotype with enhanced thrombosis potential [26]. Finally, elevated blood N,N,N-trimethyl-5-aminovaleric acid (TMAVA), a metabolite derived from microbial metabolism of trimethyl-lysine, is associated with incidental cardiac death and higher transplantation risk [27]. TMAVA may contribute to cardiac dysfunction by impairing cardiac carnitine metabolism and fatty acid oxidation, enhancing myocardial lipid accumulation and oxidative stress [27].

Periodontitis is also a risk factor for cardiovascular diseases, including peripheral artery disease, atherosclerosis, heart failure and stroke [28]. Periodontitis can lead to systemic inflammation by facilitating the translocation of periodontal pathogens such as *Porphyromonas gingivalis* from the oral cavity to the bloodstream, and *P. gingivalis* is commonly detected in human atherosclerotic plaques [29]. *P. gingivalis* may promote inflammation via TLR-NF-κB signalling [30] but also through the release of the enzyme *P. gingivalis-*derived peptidyl arginine deiminase (PPAD). PPAD catalyses the conversion of arginine into citrulline, which can act as a substrate for citrullination, a form of post-translational modification. Citrullination can lead to antigen conformational changes exposing PAMPs or inducing the generation of anti-citrullinated antibodies that contribute to autoimmune diseases such as rheumatoid arthritis [31]. While citrullinated proteins are enriched in atherosclerotic plaques [32], their role in CVD is less defined compared with autoimmune diseases.

Finally, while secondary bile acids have been shown to affect the immune response and could play a role in vascular calcification in patients with chronic kidney disease [33], a study found no association between deoxycholic acid (DCA) and atherosclerosis but did find an association between DCA and all-cause mortality and end-stage kidney disease [34].

## 3. Metabolic and Liver Diseases

Obesity and metabolic diseases are both correlated with changes in the gut microbiota composition. Landmark work from the team of Jeffrey Gordon further showed that the colonisation of germ-free mice with gut microbiota from discordant human twins could transfer the obesity phenotype [35]. While others have also confirmed that the gut microbiota composition are shifted in obesity and in metabolic diseases, there is still no clear understanding of how the gut microbiota perpetuate or contribute to metabolic alteration. The gut microbiota can affect the energy extraction efficacy from diet [35] and advances in multi-omics have identified a growing list of bacterial by-products associated with metabolic diseases.

Several lines of evidence suggest that chronic (low-grade) systemic inflammation plays a critical role in the development of metabolic syndrome. Gut epithelial disruption can increase the translocation of bacterial products, such as lipopolysaccharide (LPS), leading to endotoxemia and systemic inflammation. Subsequently, this results in insulin resistance and adipose tissue dysfunction. Patients with intestinal barrier dysfunction have increased circulating LPS-positive bacterial extracellular vesicles [36]. Increased translocation of microbiota-derived extracellular vesicles (MEV) has also been reported in obesity or type 2 diabetes mellitus patients, which has led to increased transfer of microbial DNA to pancreatic β cells [37], liver, skeletal muscle and epididymal fat [38], resulting in inflammation, β cell dysfunction and insulin resistance. Microbial DNA from MEV also trigger adrenomedullary dysfunctions and hypertension in obese mice [39]. It is interesting to note that MEV derived from specific bacteria can elicit a much stronger impact on the host’s metabolism, with the administration of *Pseudomonas panacis*-derived EV promoting glucose intolerance in mice [40].

Obesity and metabolic diseases are associated with the development of non-alcoholic fatty liver disease (NAFLD) and similar dysbiotic properties are reported across these diseases, including increased blood levels of circulating branched-chain amino acids (BCAA) and aromatic amino acids such as phenylacetic acid. In liver steatosis, these amino acids were shown to be microbiota-derived with the faecal transplantation of gut microbiota from a patient with steatosis into mice, increasing circulating BCAA (valine) and phenylacetic acid levels. This was shown to be associated with increased liver triglyceride levels within 2 weeks post faecal transplantation [41]. The effect of phenylacetic acid seems direct, as its addition to primary human hepatocytes in vitro resulted in increased triglyceride accumulation, fibrosis and reduced response to insulin, as indicated by lowered AKT phosphorylation [41]. Other by-products of amino acid metabolism can also impair host metabolism. Elevation of 3-(4-hydroxyphenyl)lactate, a by-product of microbial aromatic amino acid metabolism, was shown to be associated with hepatic steatosis and fibrosis and was significantly higher in individuals with NAFLD [42]. Furthermore, patients characterized with a ‘high fat’ liver had greater faecal levels of the lysine and histidine degradation products saccharopine and N-omega-acetylhistamine [43]. While the role of these products in liver disease are yet to be delineated, it highlights that the products of microbial amino acid metabolism are altered in NAFLD patients, and that they may serve as useful biomarkers for this disease.

TMAVA, derived from the precursor trimethyllysine, has also been found to be increased in the plasma of patients with liver steatosis [44]. Supplementation of TMAVA to mice drives early liver steatosis, characterized by increased plasma and hepatic triglyceride content as well as increased lipid accumulation in the liver [44]. TMAVA elicits these effects by inhibiting hepatic carnitine biosynthesis and fatty acid oxidation by competitively binding to γ-Butyrobetaine hydroxylase with γ-Butyrobetaine [44]. Notably, TMAVA is also a precursor for TMAO, with some evidence suggesting that TMAO could also be involved in liver disease. Several studies have reported an association of TMAO levels with triglyceride accumulation as well as with the severity of NAFLD [45]. However, its contribution to NAFLD pathogenesis or severity remains unclear, with one preclinical study demonstrating a beneficial effect of TMAO on the progression of non-alcoholic steatohepatitis (NASH) [46].

The increased production of endogenous ethanol by the gut microbiota may also contribute to NASH development. Ethanol causes oxidative stress and is mostly metabolised into acetate in the liver, providing a source of acetyl-CoA that drives de novo lipogenesis [47] and consequently steatosis. Increased concentration of ethanol has been detected from the exhaled breath of obese mice in the absence of ethanol ingestion, and is attributed to the gut microbiota [48]. NASH patients also exhibit significantly elevated blood ethanol levels, though obese individuals without NASH do not [49]. The same trend has also been observed in the faeces of children with NAFLD [50], which excludes exogenous ethanol consumption. NASH and NAFLD patients are characterized by significantly higher abundance of ethanol-producing microbes, such as *Escherichia* and *Klebsiella pneumoniae* [49,51]. The transfer of high ethanol-producing strains of *K. pneumoniae* into mice could cause NAFLD [51]. Further, alcohol dehydrogenase inhibitors induced a 15-fold increase in blood ethanol levels in patients with NAFLD, but this effect was abrogated when individuals were treated with antibiotics [52]. Altogether, gut microbiota-derived ethanol is one factor that strongly correlates with liver disease pathogenesis.

## 4. Allergic Diseases

While there is strong evidence for dysbiosis and the establishment of allergic sensitization, there is limited knowledge on the extent to which microbial-derived metabolites may contribute to or aggravate allergy development. Neonates classified as high-risk for developing atopy and asthma at 2 and 4 years had significant enrichment of faecal 12,13 DiHOME, stigma- and sito-sterols, 8-hydroxyoctanoate, α-CEHC and γ-tocopherol [53]. Similarly, increased faecal concentration of 12,13-diHOME, a linoleic acid metabolite, has been associated with increased susceptibility to atopy, eczema or asthma during childhood [54]. While SCFA offers protection from allergic diseases in mice [6,55], children with a dysfunctional gut microbiome from the age of 3 months, characterised by the depletion of 40% of bacteria specialised in the generation of SCFA, were more likely to develop atopy [56,57], suggesting that not only are SCFA beneficial but that decreased levels of SCFA could be a risk factor for allergic diseases.

Gut-derived MEV are also altered in allergic patients. The abundance of EV produced by gut bacteria of the genus *Bacteroidota* is significantly increased in the blood of asthmatic patients compared with healthy participants [58]. Another study has shown the dominance of *Bacillota*-derived EV in the urine of allergic airway children [59]. How and whether MEV contribute to asthma pathogenesis is unclear, however, EV derived from the pathogenic bacteria *Staphylococcus aureus* has been shown to trigger atopic dermatitis-like skin inflammation [60] as well as α-hemolysin-induced barrier disruption in mice [61].

## 5. Autoinflammatory Diseases

### 5.1. Inflammatory Bowel Disease and Psoriasis

Inflammatory bowel disease (IBD) development is highly linked to the gut microenvironment, with beneficial metabolites such as SCFA attenuating the inflammatory response and protecting against IBD in mice [7]. Indeed, SCFA levels have been found to be lower in both Crohn’s disease and ulcerative colitis patients [62]. Microbial-derived metabolites may also aggravate IBD. Altered microbial sulphur metabolism and increased faecal sulphated compounds, such as tauro-conjugated bile acids and glycocholic acid, are associated with IBD [63]. *Desulfovibrio,* a gut bacteria enriched in IBD patients [64], is specialised in sulphate metabolism and produces hydrogen sulphide (H_2_S) which is toxic at high concentrations. H_2_S can inhibit Acyl-CoA dehydrogenase in colonocytes which impairs butyrate oxidation, depriving epithelial cells from their major source of energy and inducing oxidative stress [65]. Furthermore, microbial H_2_S has mucolytic properties and can reduce sulphide bonds of mucus structures. This mechanism results in gut integrity degradation, which promotes hyperproliferation of epithelial cells [66]. Thus, high concentrations of H_2_S may directly contribute to the pathology of IBD by disrupting the normal physiology of colonocytes. 

Interestingly, sulphate reduction produces acetate as an end-product [67], a beneficial metabolite for IBD. Thus, the association between concentrations of acetate and H_2_S must be considered to define a state of dysbiosis that is predisposed to IBD. It is likely that the level of acetate produced via this pathway does not reach the threshold required for protection. It could also be that acetate has mostly preventive rather than therapeutic effects, or is beneficial when *Desulfovibrio* and other sulphate-reducing bacteria are low, while potentially detrimental once IBD has been established and H_2_S levels are high [67].

The process of sulphate reduction requires the presence of electron donors, and microbial-derived lactate is a major exogenic electron donor. While lactate is typically considered anti-inflammatory, its presence in an environment with high sulphide-reducing bacteria could exacerbate IBD. Indeed, higher levels of lactic acid have been demonstrated in patients with ulcerative colitis and Crohn’s disease [68,69]. It appears that lactate may be detrimental in chronic inflammatory diseases, by reducing the mobility of effector T cells to entrap them at the site of inflammation and by directly upregulating IL-17 production, as seen in rheumatoid arthritis [70,71] and, similarly, in peritonitis [72]. However, lactic acid is typically also increased during inflammation due to increased utilisation of anaerobic glycolysis to generate ATP (particularly by tumour cells, coined the Warburg effect). Therefore, whether the increased lactic acid observed in IBD and other diseases is of microbial origin must first be elucidated.

Succinate is another microbial metabolite increased in IBD, with increases observed in both the plasma and intestinal tissues of patients with Crohn’s disease and ulcerative colitis. IBD has also been characterised by the elevated intestinal expression of the succinate receptor SUCNR1 [73,74]. Deletion of SUCNR1 in mice protected against 2,4,6-trinitrobenzene sulfonic acid-induced colitis [73], indicating the direct effect of succinate on the host, contributing to IBD. Mechanistically, SUCNR1 has been found to be associated with pro-inflammatory NF-κB and ERK signalling [74] as well as the upregulation of inflammasome components in intestinal epithelial cells. Due to the high expression of SUCNR1 on various immune cells [8], it is likely that succinate can directly influence the inflammation in IBD. Indeed, SUCNR1-expressing macrophages detect extracellular succinate when activated, leading to enhanced pro-inflammatory IL-1β expression [75]. SUCNR1 is also critical for dendritic cell functionality and migratory capacity, supporting dendritic cell-induced antigen specific CD4^+^ T cell response [76] and acting as a ”chemokine” that guides their migration into lymph nodes to induce Th17 cells in arthritis [77]. We, and others, have shown that psoriasis induction in mice leads to the higher production of succinate by the gut microbiota, which induces the proliferation of resident colonic macrophages and increased levels of TNF in the gut [78,79]. Higher succinate and TNF levels in the colon have also been reported in human psoriasis [80]. Colonic inflammation associated with psoriasis aggravated colitis development in mice, which is aligned with the idea that the highest risk of developing IBD is in patients with psoriasis [79]. Interestingly, psoriasis appears to be associated with a dysregulated faecal lipid profile, with higher levels of oleic acid and linoleic acid reported in mouse psoriasis [81]. Similarly, lipid profiles were disturbed in patients with lower plasma levels of microbial-derived taurochenodeoxycholic acid, deoxycholic acid glycine conjugate, chenodeoxycholic acid glycine conjugate, and L-kynurenine [82]. Pathway analysis of differentially abundant plasma metabolites from these patients revealed enhanced α-linolenic acid and linoleic acid metabolism, which may relate to the greater production of these fatty acids by the gut microbiota in psoriasis [82].

Bacterial proteolytic fermentation and their generated by-products are also increased in patients with IBD. Such by-products include p-Cresyl sulphate and 3-indoxyl sulphate, both produced at higher levels in Crohn’s disease and ulcerative colitis patients [83]. Finally, fatty acid metabolism can also be a risk factor, with sphingolipids, sphingomyelins and ceramides being increased in the stool of Crohn’s disease patients [83].

MEV can also modulate IBD development, with the transfer of MEV derived from the gut microbiota of healthy mice protecting mice from DSS colitis. The benefits were derived via the changes in gut microbiota composition, restoration of the gut barrier integrity via the modulation of epithelial miRNA profile and the reduction of colonic inflammation [84]. Among the beneficial MEV, *Clostridium butyricum*-derived EV are protective against DSS-induced colitis by programming macrophages towards an M2 phenotype [85]. Dysbiotic MEV can also contribute to IBD by activating host TLR, leading to inflammatory responses [86]. They may also contribute to DSS colitis in a dose-dependent manner. We found that a high-protein diet increased the generation of gut MEV, which aggravated DSS-induced colitis in mice [87]. The mechanism likely involves TLR activation and NF-κB signalling contributing to pro-inflammatory responses.

### 5.2. Multiple Sclerosis

The occurrence of autoimmune diseases depends in part on a dysregulation of Treg cells, whose functions are heavily regulated by metabolites of the gut microbiome, particularly SCFA [88,89]. Alterations in the composition of the gut microbiota and of microbiota derived by-products have been reported in people with MS [90]. 

Patients with relapsing remitting MS (RRMS) had higher levels of p-Cresyl sulphate, indoxyl sulphate and N-Phenylacetyl-glutamine compared with patients treated with dimethyl fumarate. Increased levels of these metabolites correlated with a higher degree of neurodegeneration [91] as well as progression of MS [92]. Supplementation of these metabolites directly onto neuronal cell cultures induced neurotoxic effects, causing axonal damage and affected neuronal signalling [91]. Supplementation of primary oligodendrocyte progenitors with p-Cresyl reduced myelin gene expression and may contribute to myelin sheath degradation [93]. p-Cresyl sulphate might also exacerbate MS due to its structural similarities to myelin basic protein and have similar reactivity with MBP peptide 83–89 to immunoreactivity assays [92]. As discussed in the cardiovascular section, periodontitis is a risk factor for autoimmune diseases due to the citrullination of host protein by *P. gingivalis-*derived PPAD. The resulting autoantibodies directed against these proteins contribute to the development of rheumatoid arthritis, and the same mechanism could also potentiate other autoimmune diseases including MS. A recent longitudinal multi-omics and mass spectrometric study of RRMS patients has identified microbiota positively or negatively correlated with degree of disability. Circulating levels of metabolites, particularly the linoleate metabolic pathway, fatty acid biosynthesis, chalcone, dihydrochalcone, 4-nitrocatechol and methionine were altered [94]. This study pinpointed a potential correlation network linking meat servings with decreased gut microbe *Bacteroides thetaiotaomicron*, increased Th17 cell and greater abundance of meat-associated blood metabolites [94].

Interestingly, microbial-derived products appear to be important regulators of brain and neuronal activity. Mice susceptible to traumatic stress had increased p-Cresyl in the prefrontal cortex and this was associated with abnormal levels of dopamine and increased dopamine D3 receptor expression [95]. 4-Ethylphenyl sulphate, derived from the microbial metabolism of L-tyrosine, was increased in a mouse model of atypical neurodevelopment, and could alter oligodendrocyte maturation, function and oligodendrocyte-neuron interactions [96]. Importantly, mice colonized with 4-ethylphenyl sulphate-producing bacteria displayed reduced myelination of neuronal axons [96]. This indicates that 4-ethylphenyl sulphate may potentially aggravate MS, however, this remains to be investigated.

### 5.3. Systemic Lupus Erythematosus (SLE)

SLE is a complex autoimmune disease that affects numerous organs and systems in the body. While the exact cause of lupus is not fully understood, it is believed to involve a combination of genetic, environmental, and immunological factors. As in other autoimmune diseases, a role for gut bacteria metabolites in the development and progression of SLE has been proposed. Gut bacteria can produce indole derivatives and LPS that can directly activate immune cells or affect immune responses through various signalling pathways. These metabolites can potentially promote inflammation and contribute to the pathogenesis of SLE.

The role of gut microbiota dysbiosis has been studied abundantly. One of the major SLE complications, lupus nephritis, has been associated with an overall 5-fold greater representation of *Ruminococcus gnavus* of the *Lachnospiraceae* family in the gut microbiome [97]. Metabolic changes associated with active SLE include higher levels of 2-hydroxyisobutyrate and glutamate and lower levels of citrate, glycerol, linoleic acid, and propylparaben in serum [98]. Serum levels of 2-hydroxyisobutyrate are significantly higher in active patients compared with healthy individuals and inactive patients. Additionally, 2-hydroxyisobutyrate upregulation is also found in the urine of SLE patients [99].

In murine SLE models, metabolomic screening identified changes in the amount of tryptophan and derived metabolites, including higher kynurenine levels and lower 5-HT expression in the sera and faeces of triple congenic (B6.Sle1.Sle2.Sle3) lupus-prone mice compared with C57BL/6 mice. Antibiotic treatment was shown to be able to restore the faecal tryptophan of triple congenic mice to normal, and dietary tryptophan restriction exerted a protective effect on lupus progression [100]. Tryptophan metabolism could control lupus progression via multiple mechanisms. Among tryptophan-derived metabolites, indole-3-aldehyde, indole-3-acetic acid, 3-methylindole, and tryptamine, are aryl hydrocarbon receptor (AhR) ligands. AhR activation has been shown to be able to upregulate genes encoding IL-10, which regulates immune tolerance [101], so a failure to activate AhR could result in SLE exacerbation. 

## 6. Cancer

The role of the gut microbiota in cancer progression has been demonstrated by the transfer of gut microbiota from colorectal cancer patients to germ free mice, which led to accelerated tumour growth in various models [102]. Increasing numbers of gut bacteria have been identified as supporting the anti-tumour response, or as risk factors favouring tumour growth or reduced treatment efficacy, reviewed elsewhere [103]. Only a small number of bacterial metabolites have been reported to affect tumorigenesis. A study in mice has shown that diet-induced obesity exacerbated hepatocarcinoma development due to the higher generation of DCA by the gut microbiota. *Clostridium* cluster XI and XIV, which produce secondary bile acids, were enriched in these obese mice and antibiotic treatment or inhibition of the enzyme 7a-dehydroxylase, which metabolises bile acids into DCA, reversed the phenotype. DCA were found to be pro-tumorigenic by accelerating cell senescence. Intriguingly, these effects were observed in obese mice only, suggesting that additional factors were involved [104]. The pro-tumorigenic effect of DCA extends beyond liver cancer, as treatment of AKR/J mice with DCA aggravated colorectal cancer development after treatment with azoxymethane, in this cancer resistant strain [105]. While gut microbiota derived metabolites can modulate tumour development, more and more evidence suggest that the presence of gut bacteria within tumours, known as tumour microbiota, play a role in disease severity. For example, the presence of *Fusobacterium nucleatum* in human colorectal carcinoma tissue was reported in 13% of the biopsies studied [106]. *F. nucleatum* virulence factors Fap2, FadA, RadD and FomA contributed to tumour colonization, cancer cell proliferation, metastasis and immune evasion [107]. *F. nucleatum* is one of the most extensively studied tumour bacteria, with its presence being correlated with poor anti-tumour response and with lower anti-tumour immune response, as shown by decreased tumour infiltration by CD3^+^ T cells [106]. The colonization of mice with *F. nucleatum* led to more and larger colorectal tumours as well as a higher number of anti-inflammatory M2 macrophages [108]. However, *F. nucleatum* may also impair host’s anti-tumour immune response via the release of metabolites particularly formate and succinate. *F. nucleatum* has been shown to support metastasis through the release of formate. Experiments undertaken using Gut-on-Chip co-culture models have confirmed the pro-tumorigenic effect of *F. nucleatum* in primary human colorectal cancer cells but also increased their invasiveness capability via the release of formate. They confirmed that the pretreatment of tumour cells with formate leads to larger tumour and metastasis in administered mice and that treatment of mice with formate mimicked the pro-tumorigenic and metastatic effect of *F. nucleatum*. The mechanisms involved the activation of AhR by formate, leading to Wnt activation [109]. How the gut microbiota or tumour bacteria-derived metabolites affect tumorigenesis is still not fully understood. The development of in silico models could address this gap of knowledge in the future [107].

The gut microbiota also affects the response to therapies. Patients that do not respond to immunotherapy or chemotherapy have an altered gut microbiota composition. This effect is reversible, as faecal transplantation from melanoma patients that responded to anti-PD1 immunotherapy to non-responder patients increased their response to anti-PD1, as well as their CD8^+^ T cell activation [110]. To our knowledge, succinate is the only reported bacterial metabolite that can modulate response to immunotherapy. Succinate production by *F. nucleatum* reduced the tumour infiltration by CD8^+^ T cells in response to anti-PD1 immunotherapy in a mouse model of colorectal cancer. Succinate impaired the type I interferon response cGAS pathway, which decreased the expression of CCL5 and CXCL10 chemokines, responsible for tumour T cell infiltration. These effects have been shown to be reversible, as antibiotics treatment targeting *F. nucleatum* restores the efficacy of anti-PD1 treatment [111]. 

While the gut microbiota can affect a response to chemotherapy, whether bacterial-derived metabolites impair the response to chemotherapy remains elusive. A recent study has elegantly shown the beneficial effects of gut microbiota tryptophan metabolite indole-3-acetic acid on pancreatic cancer chemotherapy in mice directly treated with indole-3-acetic acid or with tryptophan. Levels of indole-3-acetic acid were also seen to be significantly lower in non-responder patients, suggesting an active role in the human response to treatment. Interestingly, this study shows that mice treated with tryptophan for 14 days developed worsened disease due to a direct impact of tryptophan on tumorigenesis, suggesting that dietary intervention must be strictly controlled [112].

## 7. Conclusions and Future Directions

Gut bacterial-derived metabolites are similarly complex in terms of their diversity and composition to the gut microbiome itself. This review highlights the way in which gut dysbiosis and a number of diseases are associated with dysbiotic patterns of metabolites, which has major implications for the initiation and perpetuation of diseases, as well as response to therapies (Table 1). Associating the gut microbiome with diseases has already resulted in compelling evidence for the role of the microbiome–host interaction. 

Moving forward, the next frontier in this field is to unlock the potential of bacterial metabolites for targeted therapeutic and preventive strategies. This involves a deeper understanding of how these metabolites work in specific contexts, and harnessing this knowledge to develop interventions that can manipulate or modulate these metabolites to benefit human health. In almost all cases, detrimental metabolites were identified in individuals with established diseases. The next step is to identify whether these metabolites are useful biomarkers for identifying an individual’s risk to various diseases, and whether their reduction can prevent disease development. One strategy would be to manipulate the diet of susceptible individuals to shift their gut microbiota activity away from the production of dysbiotic metabolites. This could simply involve reducing the consumption of foods containing high levels of L-carnitine or L-tyrosine, for example. However, the role of the gut metabolome on health is complex and will likely involve the alteration of more than one metabolite. A more appropriate strategy would involve interventions that simultaneously promote the increase of beneficial metabolites and the decrease of detrimental metabolites. Diet is the biggest lifestyle factor that defines gut microbiota composition and provides the substrates for microbial metabolism. However, the efficacy of specific diets in changing the gut microbiome is dependent on an individual’s existing gut microbiota composition and the metabolic enzymes they possess, and whether diet-induced changes persist in the long-term, both during and after cessation of the diet, remains to be demonstrated [115]. Supplements, such as probiotics and prebiotics, have similar shortfalls. The careful consideration of the type of supplement (or diet), the disease context, and the desired outcomes may need to be considered before the intervention to increase the success of such strategies [116]. More invasive methods, such as faecal microbiota transplants, may also be appropriate, as they have been shown to stably alter the recipient’s gut microbiota composition [117]. 

Regardless, if it is found that the reduction of detrimental metabolites can indeed reduce the susceptibility, or severity to disease, then these strategies may fundamentally transform how we manage non-communicable diseases. 

## Figures and Tables

**Table 1:** List of major microbiota-derived factors associated with dysbiosis, their association with different diseases and potential pathways involved.

Microbiota-Derived Factor	Source	Disease Association/Outcome	Signalling Pathways	Ref
*Metabolites*				
Trimethylamine N-oxide	TMA derived from the metabolism of dietary choline, L-carnitine, glycine, and betaine by the gut microbiota	Risk factor for CVD	Induction of pro-inflammatory factors COX-2, IL6, E-selectin, ICAM-1 via p38 MAPK signalling in endothelial and smooth muscle cells Activates endothelial inflammatory response via endoplasmic reticulum stress and NLRP3 activation	[16,18,19,20]
		Cancer: improved checkpoint blockade in pancreatic ductal adenocarcinoma	Potentiates IFN-g response CD8+ T cell antitumour response, tumour cell pyroptosis via induction of ER stress	[21,22]
		Associated with triglyceride accumulation and NAFLD severity, beneficial in NASH progression		[45,46]
Indole-3-acetic acid	L-tryptophan metabolism	Pancreatic cancer	Induces ROS accumulation and downregulation of autophagy in cancer cells	[112]
		Altered in SLE patients	Aryl hydrocarbon receptor ligand	[100]
Indoxyl sulphate	L-tryptophan metabolism	Cardiotoxic: induces arterial calcification, impaired glucose homeostasis	Decreased GLUT1 expression	[23]
		Increased in Crohn’s and ulcerative colitis patients		[83]
		Increased in relapsing remitting MS patients	Direct supplementation with neuronal cells induces neurotoxicity, altered neuronal signalling	[91]
p-Cresyl sulphate	L-tyrosine metabolism	Cardiotoxic: induces arterial calcification, impaired glucose homeostasis	Decreased GLUT1 expression	[23]
		Increased in Crohn’s and ulcerative colitis patients		[83]
		Increased in relapsing remitting MS patients	Supplementation to neuronal cells induce neurotoxicity, altered neuronal signalling Supplementation to primary oligodendrocyte progenitors decreased myelin gene expression Similar structure to myelin basic protein	[91,92,93]
Indole-3-propionic acid	L-tryptophan metabolism	Increased blood pressure in rats	Altered metabolism of cardiomyocytes in vitro	[24]
		Inverse correlation with atherosclerosis		[25]
Phenylacetylglutamine	Phenylalanine metabolism	Associated with CVD and adverse cardiovascular events	Platelet activation via α2A, α2B, and β2-adrenergic receptors, enhancing thrombosis potential	[26]
		Increased in relapsing remitting MS patients	Supplementation with neuronal cells induces neurotoxicity and altered neuronal signalling	[91]
N,N,N-trimethyl-5-aminovaleric acid	Trimethyl-lysine metabolism	Incidental cardiac death and transplantation risk	Enhance cardiac lipid accumulation and oxidative stress via alterations in fatty acid oxidation and carnitine metabolism	[27]
		Increased in plasma of patients with liver steatosis, drives early steatosis in mice	Inhibits hepatic fatty acid oxidation and carnitine biosynthesis	[44]
Deoxycholic acid	Microbiota modification of host bile acids	Association with all-cause mortality and end-stage kidney disease		[34]
	*Clostridium* cluster XI and XIV	Exacerbated hepatocarcinoma development in obese mice	Acceleration of cell senescence	[104]
		Aggravated colorectal cancer development in AKR/J mice		[105]
Phenylacetic acids		Associated with liver steatosis	Directly increased triglyceride accumulation, fibrosis, and impaired insulin response in primary human hepatocytes	[40,41]
Valine		Associated with liver steatosis. Increased circulating levels in patient with steatosis	Increased triglyceride levels in liver in mice transplanted with steatosis microbiota	[40]
3-(4-hydroxyphenyl)lactate	Aromatic amino acid metabolism	Associated with hepatic steatosis and fibrosis. Higher in individuals with NAFLD		[42]
Saccharopine	Lysine metabolism	Increased faecal levels in patients with “high fat” liver		[43]
N-omega-acetylhistamine	Histidine metabolism	Increased faecal levels in patients with “high fat” liver		[43]
12,13 DiHOME	Linoleic acid metabolite	Enriched in faeces of neonates at high risk for atopy and asthma		[53]
		Associated with increased susceptibility to atopy, eczema and asthma in childhood		[54]
Propionate		Beneficial in MS patient relapses, but may promote inflammation in blood brain barrier	Restoration of Treg/Th17 balance	[113,114]
2-hydroxyisobutyrate		Increased in active SLE patients		[97,98,99]
Hydrogen sulphide	*Desulfovibrio*	Enriched in IBD patients	Inhibits colonocyte metabolism, inducing oxidative stress in epithelial cells. Impairs mucus layer via reduction of sulphide bonds in mucus, triggering epithelial cell hyperproliferation	[64,65,66]
Lactate	Microbiota or host	Increased in Crohn’s and ulcerative colitis patients	Electron donor for sulphate reduction, exacerbating effects of sulphide-reducing bacteria	[68,69]
	Microbiota or host	Rheumatoid arthritis, peritonitis	Upregulates IL-17 production, reduces mobility of T effector cells	[70,71,72]
Succinate	Microbiota	Increased in the plasma and tissue of Crohn’s and ulcerative colitis patients	Deletion of receptor SUCNR1 is protective in murine colitis, via NF-κB, ERK signalling, inflammasome	[73,74]
		Psoriasis in humans and mice	Proliferation of resident colonic macrophages, increasing gut TNF	[78,79,80]
		Pro-inflammatory effects on immune cells	SUCNR1 activation on macrophages enhances IL-1β, supports DC antigen-specific response, induces Th17 cells	[75,76,77]
	*Fusobacterium nucleatum*	Decreased response to immunotherapy in mouse colorectal cancer	Reduced CD8+ tumour infiltration in anti-PD-1 therapy due to decreased chemokine expression via impaired cGAS pathway	[111]
Formate	*Fusobacterium nucleatum*	Pro-tumorigenic in primary human colorectal cancer cells	Activation of aryl hydrocarbon receptor triggering Wnt activation	[109]
Ethanol	Endogenous production by gut microbiota	Promotes steatosis	Metabolism to acetyl-CoA that drives de novo lipogenesis	[47]
	Increased abundance of EtOH promoting microbes *Escherichia* and *Klebsiella pneumoniae*	Elevated blood levels in NASH patients, children with NAFLD		[49,50,51]
*Enzymes, vesicles, virulence factors*				
Peptidyl arginine deiminase (PPAD)	*P. gingivalis* in oral cavity	Periodontitis risk factor for CVD, *P. gingivalis* associated with atherosclerotic plaques	TLR-NF-kB signalling	[28,29,30]
		Periodontitis risk factor for autoimmune disease, associated with rheumatoid arthritis	PPAD promotes protein citrullination, generating anti-citrullinated antibodies which contributes to autoantibodies	[31]
Microbiota extracellular vesicles (MEV)	Microbiota	May contribute to systemic inflammation in metabolic disease. Increased translocation of MEV in blood of obese and T2DM patients	Microbial DNA exposure to pancreatic β cells, liver, muscle and fat cells, causing dysfunction and insulin resistance	[37,38]
		Hypertension in obese mice	Microbial DNA triggers adrenomedullary dysfunction	[39]
	*Pseudomonas panacis*	Increased glucose intolerance in mice		[40]
	*Bacteroidetes*	Increased EVs in asthmatic patient blood		[58]
	Firmicutes	Increased EV in urine of allergic children		[59]
	*Staphylococcus aureus*	EV trigger skin inflammation in mice	Skin barrier disruption by α-hemolysin	[60,61]
	Healthy microbiota	Protective in DSS colitis	Altered microbiota composition, improved gut barrier integrity via modulating epithelial miRNA	[84]
	*Clostridium butyricum*	Protective in DSS colitis	Promote M2 macrophage phenotype	[85]
	Dysbiotic microbiota, increased in high protein diet	Aggravate IBD	Activating host TLRs	[86,87]
Fap2, FadA, RadD and FomA	*Fusobacterium nucleatum*	Cancer	Virulence factors that promote tumour colonization, cancer cell proliferation, metastasis and immune evasion	[106,107]

## Data Availability

Not applicable.
